# Monkeypox: A Comprehensive Review

**DOI:** 10.3390/v14102155

**Published:** 2022-09-29

**Authors:** Harapan Harapan, Youdiil Ophinni, Dewi Megawati, Andri Frediansyah, Sukamto S. Mamada, Mirnawati Salampe, Talha Bin Emran, Wira Winardi, Raisha Fathima, Salin Sirinam, Pichamon Sittikul, Ana M. Stoian, Firzan Nainu, Malik Sallam

**Affiliations:** 1 Medical Research Unit, School of Medicine, Universitas Syiah Kuala, Banda Aceh 23111, Indonesia; 2 Tropical Disease Centre, School of Medicine, Universitas Syiah Kuala, Banda Aceh 23111, Indonesia; 3 Department of Microbiology, School of Medicine, Universitas Syiah Kuala, Banda Aceh 23111, Indonesia; 4 Tsunami and Disaster Mitigation Research Center (TDMRC), Universitas Syiah Kuala, Banda Aceh 23111, Indonesia; 5 Ragon Institute of MGH, MIT and Harvard, Cambridge, MA 02139, USA; 6 Laboratory of Host Defense, WPI Immunology Frontier Research Center (IFReC), Osaka University, Osaka 565-0874, Japan; 7 Department of Veterinary Pathobiology, School of Veterinary Medicine, University of Missouri, Columbia, MO 65211, USA; 8 Department of Microbiology and Parasitology, School of Medicine, Universitas Warmadewa, Bali 80239, Indonesia; 9 Research Group for Food Microbiology and Biotechnology, National Research and Innovation Agency (BRIN), Yogyakarta 55861, Indonesia; 10 Department of Pharmacy, Faculty of Pharmacy, Hasanuddin University, Makassar 90245, Indonesia; 11 Sekolah Tinggi Ilmu Farmasi Makassar, Makassar 90242, Indonesia; 12 Department of Pharmacy, BGC Trust University Bangladesh, Chittagong 4381, Bangladesh; 13 Department of Pharmacy, Faculty of Allied Health Sciences, Daffodil International University, Dhaka 1207, Bangladesh; 14 Department of Pulmonology and Respiratory Medicine, School of Medicine, Universitas Syiah Kuala, Banda Aceh 23111, Indonesia; 15 Department of Tropical Pediatrics, Faculty of Tropical Medicine, Mahidol University, Bangkok 10400, Thailand; 16 Department of Medical Microbiology and Immunology, School of Medicine, University of California Davis, Davis, CA 95616, USA; 17 Department of Pathology, Microbiology and Forensic Medicine, School of Medicine, The University of Jordan, Amman 11942, Jordan; 18 Department of Clinical Laboratories and Forensic Medicine, Jordan University Hospital, Amman 11942, Jordan; 19 Department of Translational Medicine, Faculty of Medicine, Lund University, 22184 Malmö, Sweden

**Keywords:** public health, emergency, international concern, zoonosis, emerging viral infectious diseases, epidemic, vaccination, prevention

## Abstract

The 2022 multi-country monkeypox outbreak in humans has brought new public health adversity on top of the ongoing coronavirus disease 2019 (COVID-19) pandemic. The disease has spread to 104 countries throughout six continents of the world, with the highest burden in North America and Europe. The etiologic agent, monkeypox virus (MPXV), has been known since 1959 after isolation from infected monkeys, and virulence among humans has been reported since the 1970s, mainly in endemic countries in West and Central Africa. However, the disease has re-emerged in 2022 at an unprecedented pace, with particular concern on its human-to-human transmissibility and community spread in non-endemic regions. As a mitigation effort, healthcare workers, public health policymakers, and the general public worldwide need to be well-informed on this relatively neglected viral disease. Here, we provide a comprehensive and up-to-date overview of monkeypox, including the following aspects: epidemiology, etiology, pathogenesis, clinical features, diagnosis, and management. In addition, the current review discusses the preventive and control measures, the latest vaccine developments, and the future research areas in this re-emerging viral disease that was declared as a public health emergency of international concern.

## 1. Introduction

A multi-country outbreak of human monkeypox was reported by the World Health Organization (WHO) in May 2022 [1]. Since the start of this outbreak, a cumulative total of 64,290 laboratory-confirmed monkeypox cases have been reported in 106 countries across the globe, with 20 deaths as a result of the disease as of 21 September 2022 [2]. The swift pace of the outbreak has brought a new public concern on the rise of another viral pandemic and public health threat [3].

The causative agent of monkeypox disease, monkeypox virus (MPXV), has been identified for more than 60 years [4]. In 1959, the first description of monkeypox was published in a report that described two outbreaks of pox-like disease during the summer and autumn of 1958, among *Macaca fascicularis* monkeys housed in Statens Serum Institut in Copenhagen, Denmark [5]. These outbreaks were attributed to a newly described poxvirus, which was then named monkeypox. Subsequently, several monkeypox outbreaks have been identified in laboratories or zoos among captive monkeys [6]. In 1970, human infection by MPXV was first recognized in a 9-month-old child in the Democratic Republic of the Congo [7]. Since then, monkeypox has been reported as a zoonosis endemic in Central and Western Africa [8,9]. Prior to the current 2022 outbreak, human-to-human transmission of MPXV had been reported in endemic countries in Central Africa [10]. Additionally, monkeypox outbreaks have also been reported in non-endemic countries, which were mostly linked to imported animals from the endemic regions, with the 2003 outbreak in the United States (U.S.) as a notable example [11,12]. The previous experience of monkeypox outbreaks highlighted the global relevance of this emerging zoonosis [13].

Several factors are linked to the increased frequency of monkeypox outbreaks observed in the past 40 years [13,14]. These factors include the increased susceptibility to monkeypox infection following the cessation of smallpox vaccination [14]. It has been shown that vaccination against smallpox confers about 85% effectiveness in the prevention of monkeypox [15]. An additional possible factor involves the extensive consumption of animals as a protein source which are potential MPXV reservoirs, particularly in regions afflicted by poverty and social crises such as civil wars [14]. Other factors linked to the emergence of monkeypox outbreaks include the increased population density, ease of travel, and ecological and environmental factors (e.g., clearing of tropical rainforests) with an increased risk of exposure to reservoir animals [14,16,17].

This review aims to provide a comprehensive and up-to-date overview of monkeypox, including its epidemiology, etiology and pathogenesis, clinical features, laboratory findings, complications and sequelae, management and preventive measures, including vaccination. The latest vaccine development and potential directions are also discussed.

## 2. An Updated Overview of Confirmed Case Numbers Worldwide

As of 21 September 2022, the total number of confirmed monkeypox cases that were recorded during the ongoing outbreak was 64,290 across 106 countries worldwide [2]. Of these cases, 579 were confirmed in seven endemic countries with a previous history of monkeypox cases [2].

**Figure 1:**
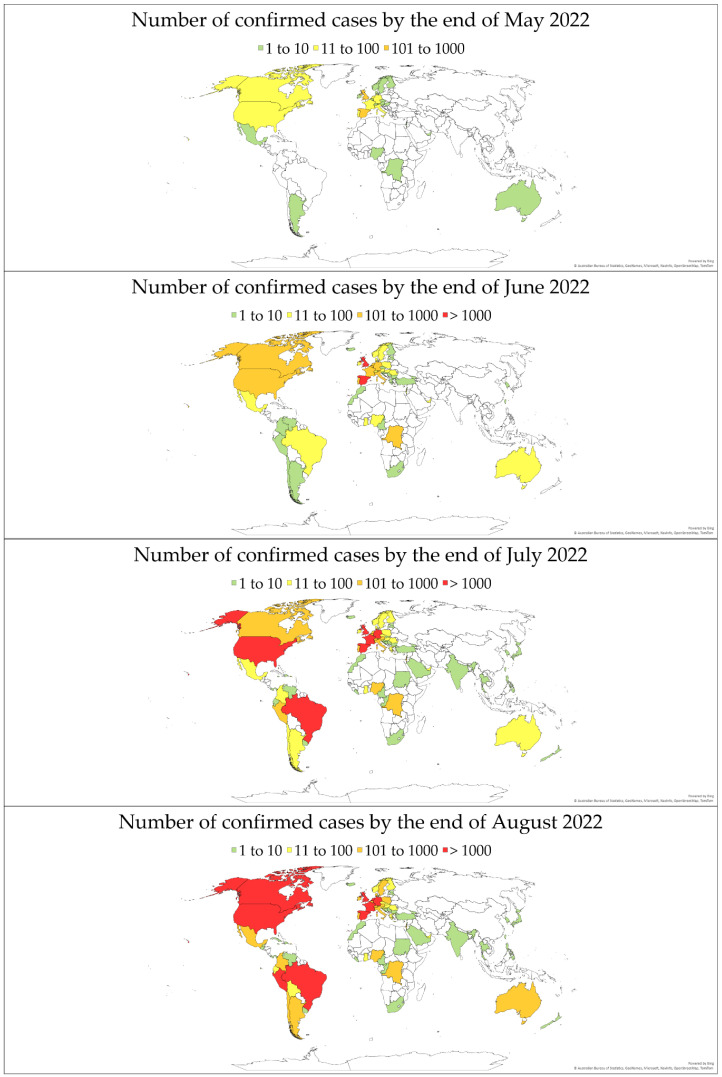
The total number of confirmed monkeypox cases per country. Data are based on (OurWorldInData.org, accessed on 9 September 2022) as reported by the end of May, June, July, and August 2022 [18].

The evolution of monkeypox cases by the end of each month is illustrated in Figure 1, using data from (OurWorldInData.org, accessed on 9 September 2022) [18].

## 3. Virology and Genomic Classification

The monkeypox virus belongs to the same group as variola, cowpox, and vaccinia viruses classified within the genus *Orthopoxvirus*, family *Poxviridae*. One unique trait of MPXV among other poxviruses is the broad range of host species tropism—from rope squirrels to sooty mangabey [19,20]—which may have allowed prolonged zoonotic circulation of MPXV in the wild. The name ‘monkeypox’ was coined after the first isolation in 1958 from infected cynomolgus monkeys [5]. However, this name might be a misnomer as (1) serological evidence from animal samples seems to point to rodents as the primary natural reservoirs, while infections in primates are merely spillover events [21,22], and (2) direct transmission may also occur between spillover hosts other than monkeys, e.g., human-to-human, particularly in light of the latest global outbreak in 2022. Indeed, phylogenetic analyses from recent cases also indicate genomic separation of the new isolates from the original monkey-infecting MPXV strains [23]. These reasons are factored into the current discussion for a new name for MPXV [24]. Nevertheless, inoculation of MPXV into nonhuman primates has proven to be an ideal animal infection model for poxvirus, as it causes nearly identical symptoms to smallpox infection in humans, albeit milder and with a lower transmission rate [25]. Smaller animal models are also being investigated, including BALB/c mice [26].

**Figure 2:**
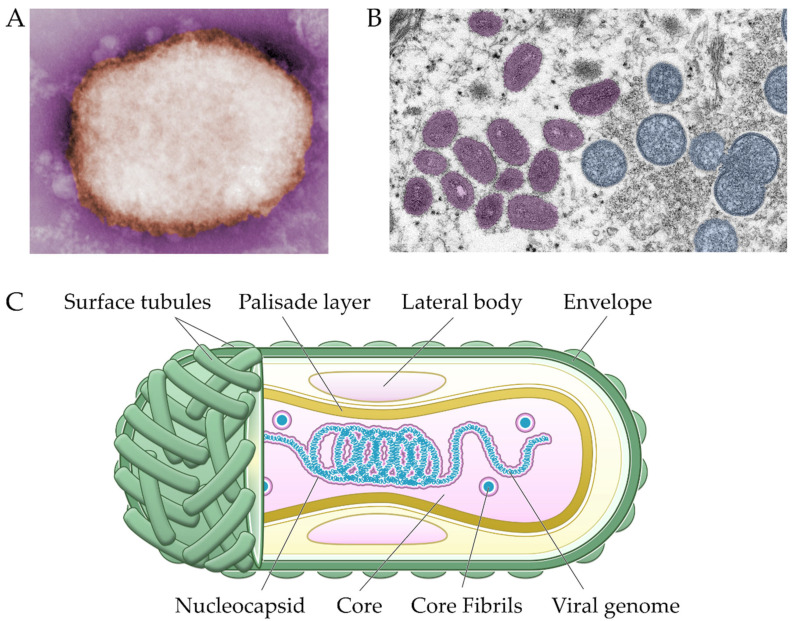
Negative-stained transmission electron micrograph of M-type MPXV particle (**A**). Thin section of viral particles on skin sample, showing ovoid mature virions on the left and spherical immature virions on the right. Micrographs are courtesy of the Centers for Disease Control and Prevention (CDC) Public Health Image Library (PHIL) [28] (**B**). Schematic representation of MPXV virion structure (**C**).

Morphologically, MPXV exhibits an ovoid or rectangular brick-shape characteristic of poxviruses, measuring 200 × 250 nm, decorated with membrane surface tubules or filaments and a biconcave core component as seen on electron micrograph (Figure 2) [27].

Immature virion can be discerned by its more spherical shapes, while mature virion can be seen in negative staining in two forms: mulberry (M), which is smaller with short (10 nm) surface tubules; or capsular (C), which is slightly larger and penetrated by staining, thus showing multiple laminated zones. The life cycle of poxviruses is unique among DNA viruses: viral replication is exclusively restricted in the cytoplasm without interfering with the host genome. The MPXV genome is a linear double-stranded DNA with a massive length of over 197 kb consisting of around 200 genes, which presents a considerable challenge during de novo whole-genome assembly [29]. All proteins necessary for replication and structural assembly are encoded within the viral genome, covalently closed at both ends by two inverted terminal repeats (ITRs) of around 10 kb each. Typical of orthopoxviruses, sequence conservation is high in the central region of the MPXV genome but decreases toward the terminal ITRs. Here, the genes responsible for housekeeping are located in the central region and thus are highly conserved among orthopoxviruses, while genes encoding proteins that interact with host factors have lower sequence identity and are located further toward the termini regions [30,31]. The latter coding genes are aptly named virulence factors, as most seem to be dispensable for in vitro replication in cell culture, but their absence attenuates in vivo pathogenesis [32].

Based on the sequence identity of all strains isolated from the African continent, MPXV can be differentiated into two clades: strains isolated from West Africa and from the Congo Basin (or Central Africa), where inter-clade sequence homology is at most ~95% while intra-clade homology approaches 99% [33]. Besides geographic distribution, these two clades vary in clinical presentation, severity, and transmission [4]. The West African clade appeared milder, without reported mortalities until the 2017–2018 Nigeria outbreak, whereas the case–fatality ratio for the Congo Basin clade was reported to be approximately 10% [9,34]. The preliminary phylogenetic investigation revealed that the current 2022 outbreak is mainly related to the West Africa clade [35].

**Figure 3:**
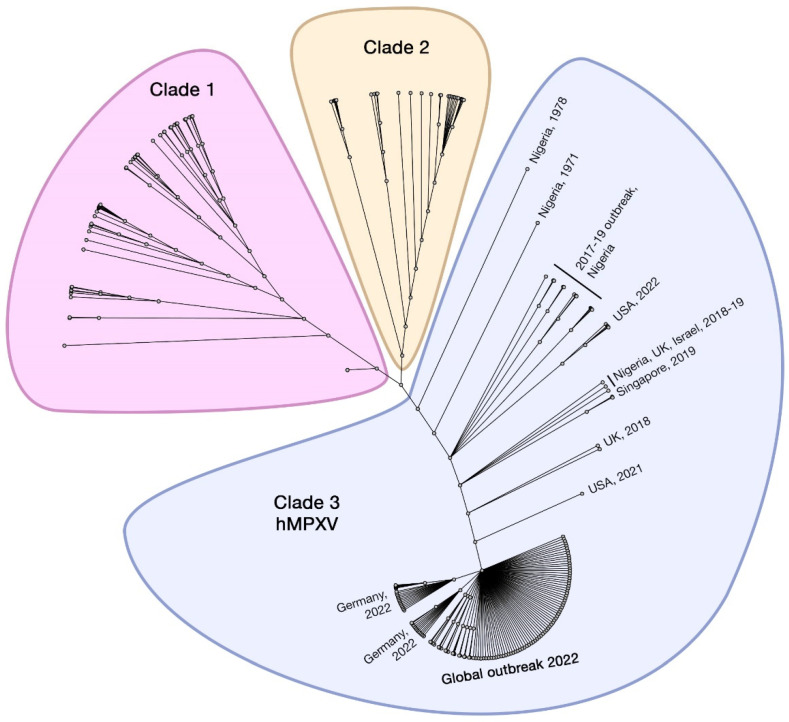
Unrooted phylogenetic tree of MPXV genomes from 219 isolates sampled from 1970–2022 using iqtree2, aligned to reference (NC_063383) at the 3′ inverted terminal repeat (ITR) region using Geneious Prime. The tree shows the recently proposed three-clade classifications, consisting of Clade 1 (previously known as Congo Basin/Central Africa), Clade 2 (Western Africa), and Clade 3 (strong evidence of human-to-human transmission of MPXV (hMPXV)).

A change in the nomenclature system of MPXV clades has been considered to avoid discriminatory geographical identification. A fitting example was established by Happi et al. [24,36], where the isolates sourced from the Congo Basin are noted as MPXV Clade 1, and those rooted in West Africa are noted as Clade 2 and 3. Here, we adopt this three-clade classification (Figure 3). Notably, virulence differs between the clades: Clade 2 and 3 are less virulent and less transmissible in humans and non-human primates (NHPs) than Clade 1. Such findings may explain the zero-case fatality in the 2003 outbreak in the U.S. [12]. In addition, 90% of reported cases hailed from the Congo Basin rather than outside it, despite similar nonvaccinated seroprevalence between both regions [37]. However, the 2022 outbreak shows signs of separation from the original two clades, particularly in the transmission efficiency between humans; this subclade branched from Clade 2 and is currently noted as Clade 3 or ‘human MPXV’ (hMPXV).

The highest diversity between Clade 1 and 2 (and 3) seems to be clustered in the terminal regions toward the ITRs, which contain genes encoding host-response modifier (HRM) proteins. One of these is the monkeypox ortholog of the poxviral inhibitors of complement enzymes (PICEs) or the MOPICE protein, which was once thought of as the differential virulence factor between Clade 1 and 2, i.e., lack of MOPICE in Clade 2 contributes to its lesser pathogenicity [33,38]. However, a robust study in rhesus macaques showed the opposite: MOPICE deletion increased in vivo replication and weakened adaptive immune response [39]. Virulence determinants differentiating the two clades seem to be more likely influenced by many genetic factors within the massive genome of MPXV, including the open reading frames of D10L, B10R, B14R, and B19R [40]. Another consideration is whether MPXV virulence correlates with genetic variability across MPXV clades or is independent from these viral factors. The recent 2022 outbreak, however, might indicate that the former is true; all global isolates are phylogenetically derived from Clade 2, and to date, only 20 deaths has been reported among over 60,000 laboratory-confirmed cases [2,41].

## 4. Pathophysiology and Immune Evasion

The MPXV uses several entry routes to enter the human body, such as oropharyngeal, nasopharyngeal, or intradermal routes [42]. Interestingly, it has also been found that MPXV could gain entry into the body through sexual transmission [43,44]. Transmission occurring among humans can go through direct contact with an infected skin lesion or mucosa, or droplets from breathing [43,44,45]. Moreover, direct contact with materials contaminated with the virus, such as clothes, utensils, and furniture, is also considered [43,44].

Following entry, the virus replicates at the inoculation site and spreads directly to the local lymph nodes [42]. After its incubation period (1–3 weeks), several symptoms appear, e.g., backache, sore throat, shortness of breath, fever, chills, malaise, headache, and enlarged lymph nodes [46,47]. Approximately 1–3 days after the appearance of fever and lymphadenopathy, the patient enters the infectious stage represented by the development of a rash that often appears first in the facial area and then spreads to other parts of the body [46,48].

Like other viruses, the genus of *Orthopoxvirus* has developed various mechanisms to evade the host’s defense systems. This ability facilitates the entry of the virus without being detected or recognized by the systems. Some of those mechanisms are described briefly below.

**Figure 4:**
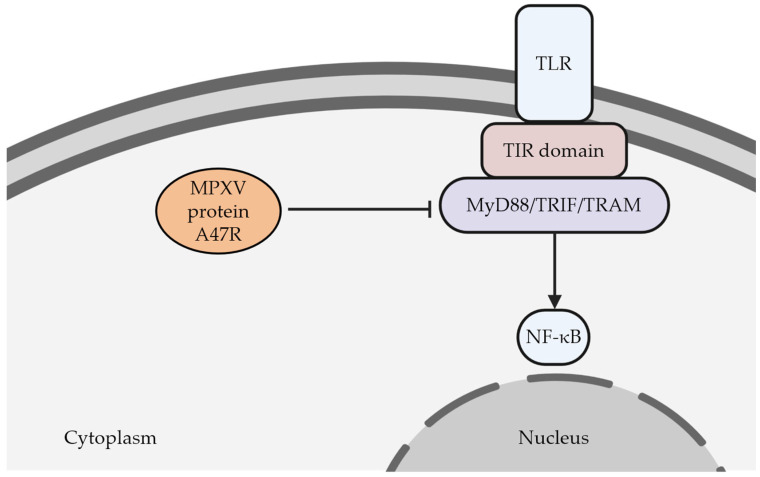
As a member of pattern recognition receptors (PRRs), TLR plays a critical role in recognizing various noxious molecules. Upon its interaction with those molecules, the cytoplasmic domain (TIR) of TLR recruits the appropriate adaptor proteins (e.g., MyD88, TRIF, or TRAM). This interaction induces the subsequent molecular pathways that will eventually upregulate the expression of NF-κB, which is critical in modulating the innate immune system. It has been found that this mechanism can be impaired by the action of a MPXV protein called A47R. This viral protein can interact with the adaptor proteins leading to the impairment of viral recognition by the immune system. TLR (Toll-like receptor); TIR (Toll/interleukin-1 receptor); MyD88 (myeloid differentiation primary-response gene 88); TRIF (TIR-domain-containing adaptor protein inducing IFNβ); TRAM (TRIF-related adaptor molecule); NF-κB (nuclear factor kappa B). The figure was created by Biorender.

It has been demonstrated that orthopoxviruses can disturb the pattern recognition receptors (PRRs) expressed by the innate immune cells. These proteins consist of several subfamilies, including the Toll-like receptors (TLRs), NOD-like receptors (NLRs), RIG-1-like receptors (RLR), and C-type lectin receptors (CLRs). They are responsible for recognizing various microbe-related molecules or molecules released by impaired cells [49]. Once the PRRs bind to the microbial ligands, the subsequent cascades occur, including activation of inflammation-related transcription factors such as nuclear factor kappa B (NF-κB), interferon regulatory factors (IRFs), and activating protein-1 (AP-1) [50]. It has been known that signal transduction of TLRs involves several types of intracellular adaptors proteins such as MyD88, MAL, TRAM, TRIF, and SARM, which are pivotal for triggering intracellular immunologic reactions [49,51]. Any disruption in those adaptor proteins may cause problems in exerting adequate immunologic response towards viral infections. At this point, orthopoxviruses contain genes encoding proteins that could interact and damage the functionalities of those adaptor proteins. For example, MPXV could produce a protein called A47R, which can interact with MyD88, TRIF, and TRAM [50] (Figure 4). Consequently, these adaptor proteins’ physiological functions are disturbed, followed by the inhibition of the transcription factors associated with inflammation, i.e., NF-κB [50]. Ultimately, this condition leads to the failure of the innate immune systems to recognize the viruses.

The development of viral proteins showing properties as apoptosis inhibitors becomes another strategy utilized by orthopoxviruses, including MPXV, to evade the host’s defense systems [50]. Apoptosis is a common and essential mechanism found in multicellular organisms to prevent viral proliferation and diminish the spread of infection to the other cells by killing the infected cells. As mentioned above, the ability of MPXV to inhibit NF-κB activity results in the failure of the immune system to recognize the virus. It has been clearly documented that NF-κB also plays a fundamental role in regulating apoptosis [52,53].

In addition to the disruption of NF-κB regulation, other specific mechanisms are also proposed to explain the ability of *Orthopoxviruses* to inhibit cellular apoptosis. It has been reported that orthopoxviruses, including MPXV, might hinder the activities of caspase-1, caspase-8, and caspase-9, which are essential in executing apoptosis [50,54]. In MPXV strain Zaire-I-96, this inhibitory action might be mediated by several viral proteins (e.g., B12R and C7L) [50].

Furthermore, like other orthopoxviruses, MPXV also has a gene that encodes protein mimicking activity of Bcl-2 proteins, which have been known to play a critical role in regulating apoptosis [50,55]. The viral protein P1L has been revealed to have activity similar to B-cell lymphoma-2 (Bcl-2)-like proteins in MPXV strain Zaire-I-96 [50]. Molecularly, this viral protein interacts with the IκB kinase (IKK) complex, which is vital for facilitating the activation of NF-κB [50,56,57]. This action also leads to the failure to induce cellular apoptosis. It has also been demonstrated that MPVX and other orthopoxviruses could produce proteins acting as an inhibitor of interferon, which is pivotal in tackling viral infection. This activity is mediated by the ability of MPVX to block the production of interferon regulatory factors (IFRs), which are known as the initial cascade in interferon production [50].

In addition to the mechanisms mentioned above, orthopoxviruses have other multiple genes encoding proteins used to perturb various stages of the host’s inflammatory cascade. They could disturb the production of cytokines and chemokines, the activity of the complement system, the activity of the ubiquitin–proteasome pathway, and several other targets [50,58].

Following its success in avoiding the host’s immune system, MPXV is capable of attacking many sites within the human body. In this case, clinical manifestations of monkeypox are remarkably similar to those of smallpox. However, although these infectious diseases share many uniformities in their signs and symptoms, several manifestations are used to differentiate smallpox and monkeypox. For example, lymphadenopathy is closely associated with monkeypox but is not a characteristic of smallpox [43]. The enlargement could occur in lymph nodes located at various sites. Nevertheless, the nodes in submandibular, cervical, or inguinal areas seem to be the primary sites of MPXV-related lymphadenopathy [47].

The enlargement of lymph nodes may indicate that the immune response activated by the host following MPXV infection is more effective than the infection caused by the other orthopoxviruses [43]. To date, no clear explanation for this phenomenon has been reported. However, this might be caused by different viral proteins produced among the orthopoxviruses, and this could be seen in the case of a viral protein called the vaccinia complement control protein (VCP) produced by orthopoxviruses [50]. The VCP consists of four short consensus repeats (SCRs); each consists of approximately 60 amino acids, resembling a regulator of complement activation. VCP can bind to several complement components (e.g., C3b and C4b), followed by disturbance of the subsequent complement cascades [50]. Ultimately, VCP suppresses inflammatory response [59,60].

The intact structure of VCP protein is found in other orthopoxviruses (viruses of variola, cowpox, and vaccinia). In contrast, in MPXV, the structure of VCP is either truncated (Clade 1 or Congo Basin/Central Africa Clade) or deleted (Clade 2 or Western African Clade) [50]. The unique structure of VCP in MPXV causes the functional activity of VCP in repressing the host’s inflammatory response to be inadequate. Consequently, in MPXV infection, the immune response generated by the host is more intense, and this possibly causes the event of lymphadenitis [50].

## 5. Clinical Characteristics and Laboratory Findings

While the clinical features of monkeypox are similar to those of smallpox infection, the presence of the symptom might vary depending on the virus clades and endemic or non-endemic setting (Table 1).

One large study of serology testing during the monkeypox outbreak in Cameroon demonstrated that many individuals who did not show any symptoms had a high titer of *Orthopoxvirus* IgG and IgM antibodies detected by ELISA [65], suggesting that the infection might be asymptomatic in some populations [66].

The incubation period is reported to be around 5 to 21 days [67]. The infection can be divided into two phases: the invasion period and the skin eruption period. The invasion period mainly occurs from day 1 to day 5, characterized chiefly by chills, fever, sore throat, headache, myalgia, and lymphadenopathy [62,63,68,69]. Lymphadenopathy is a hallmark of monkeypox and is essential to distinguish it from other orthopoxviral infections, including smallpox, measles, or chickenpox. It usually occurs 1–3 days after the onset of fever and is rarely concurrent with the onset of the rash. Lymphadenopathy can occur in submandibular glands and neck, axilla, or groin. It might appear on both sides or only one side of the body and sometimes can be painful.

The skin eruption period usually occurs within 1–3 days of fever. It evolves in 1 to 2 days following the macular, popular, vesicular, and pustular phases [69]. An overlap appearance of the lesion might occur during the course. Initial lesion might start from the mouth, followed by centrifugally concentrated lesion of the face and extremities, and is characterized by 2–10 mm in size, hard, thick lesions.

Several symptoms were reported to be linked with hospitalization, such as >100 lesions, adenopathy, mouth sores, dysphagia, nausea, and vomiting. In addition, nausea and vomiting were independently associated with a longer duration of hospitalization [61].

**Figure 5:**
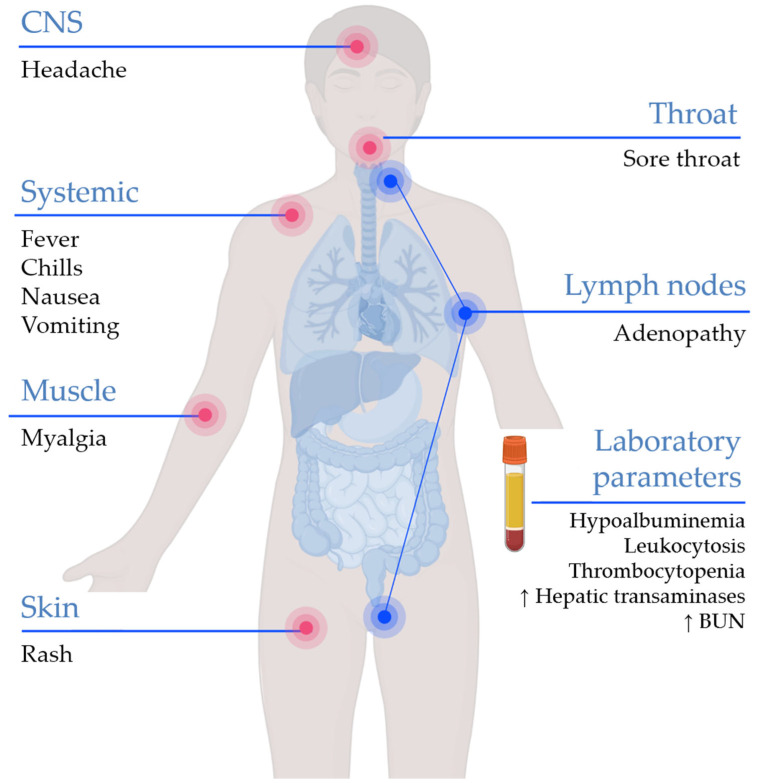
The clinical and laboratory features of monkeypox. BUN: blood urea nitrogen. The figure was created by Biorender.

Previous studies demonstrated that elevation of transaminase level, low blood urea nitrogen level, hypoalbuminemia, leukocytosis, and thrombocytopenia were the most common alterations in laboratory parameters among monkeypox-infected patients [61,62,64]. Elevation of alanine aminotransferase (ALT) and aspartate aminotransferase (AST) were reported as predictors of poor prognosis [62]. A summary of common clinical and laboratory findings of monkeypox is illustrated in (Figure 5).

## 6. Diagnosis

The evaluation of monkeypox should include specific medical history taking and clinical appearance assessment. A history of traveling to an endemic area, interacting with a wild animal from infected areas, or taking care of an infected patient should always be considered to help establish the diagnosis. However, the final diagnosis should be justified by laboratory findings. This comes in light of the long list of differential diagnoses for acute rash as the presenting complain accompanied by nonspecific symptoms (e.g., fever, headache, myalgia, asthenia, etc.) [70].

Therefore, the following conditions should be considered: varicella (chickenpox), measles, molluscum contagiosum, cutaneous bacterial infections, scabies, syphilis, and drug allergies [70,71]. One feature that can help in differentiating monkeypox from varicella and variola is lymphadenopathy during the prodromal phase [71]. In view of frequent sexual transmission observed during the 2022 outbreak, other sexually transmitted infections (STIs) should be considered as well, including herpes simplex virus infections (including eczema herpeticum and disseminated herpes virus infections in immunocompromised patients), *Haemophilus ducreyi* chancroid, *Chlamydia trachomatis* lymphogranuloma venereum (LGV), and *Klebsiella granulomatis* granuloma inguinale [70]. Other non-infectious conditions in the differential diagnoses include: Behçet’s disease, squamous cell carcinoma, and recurrent aphthous stomatitis [70].

Real-time polymerase chain reaction (PCR) is the modality of choice for laboratory tests and has been widely used to detect MPXV [48]. PCR testing is highly efficient and sensitive for detecting the presence of viral DNA from patient specimens. The specimens for the examination can be taken from the lesion exudate or scabs [72]. Other methods such as virus isolation, immunohistochemistry, IgG and IgM enzyme-linked immunosorbent assay (ELISA), and electron microscopy can also be performed, although they certainly require more sophisticated tools and specialized facilities, such as a proper biosafety level for virus handling [69].

## 7. Treatment

Monkeypox infection can be self-limiting, and supportive care is generally recommended. Individuals who have no risk of severe symptoms can remain isolated at home. Healthcare personnel should evaluate on a case-by-case basis if the infection prevention and control conditions within the home environment are met.

### 7.1. Management of Mild or Uncomplicated Monkeypox

Symptomatic relievers can be prescribed according to the patient’s condition, for example, antipyretics, analgesics, or antiemetic medication. Adequate hydration, vaccination review, and nutritional assessment should be performed, especially in pediatric patients. Supplementing vitamin A, which has demonstrated an essential role in wound healing, may benefit deficient patients [73].

Mild skin rashes can be given supportive treatment to quell irritation and promote healing. Antimicrobial agents to eradicate *Streptococcus pyogenes* or *Staphylococcus aureus* are recommended if a secondary bacterial infection is suspected. Complications such as cellulitis, necrotizing soft tissue infection, or abscess should be monitored and treated appropriately.

Mental health should also be followed up in patients with monkeypox. Long-term isolation can cause anxiety and depression, which should be helped with psychological support [74].

### 7.2. Management of High-Risk Patients and Severe or Complicated Monkeypox

The prognosis for monkeypox is determined by several factors, including age, previous vaccine history, current health status, and comorbidities. Patients with high risk for severe disease, i.e., children (especially those under eight years old, who have the highest mortality rate [16]), pregnant women [75,76], the immunocompromised, and individuals who have poor skin integrity (e.g., atopic dermatitis or exfoliative skin conditions), should be hospitalized for monitoring and considered for antiviral treatment. Confluent rashes or skin lesions of more than a hundred, based on the studies conducted on smallpox, indicate severe disease [77].

Monkeypox infection with progressive illness or complications should be treated as well as high-risk patients. Severe dehydration from gastrointestinal loss, pneumonia, encephalitis, sight-threatening ocular lesions, and sepsis can potentially occur, requiring antiviral agents and specific treatment.

### 7.3. Antiviral Agents

To date, there is no specific treatment for monkeypox. Several antiviral drugs approved for treating smallpox or other orthopoxviruses have been repurposed to manage monkeypox infection.

#### 7.3.1. Tecovirimat

**Figure 6:**
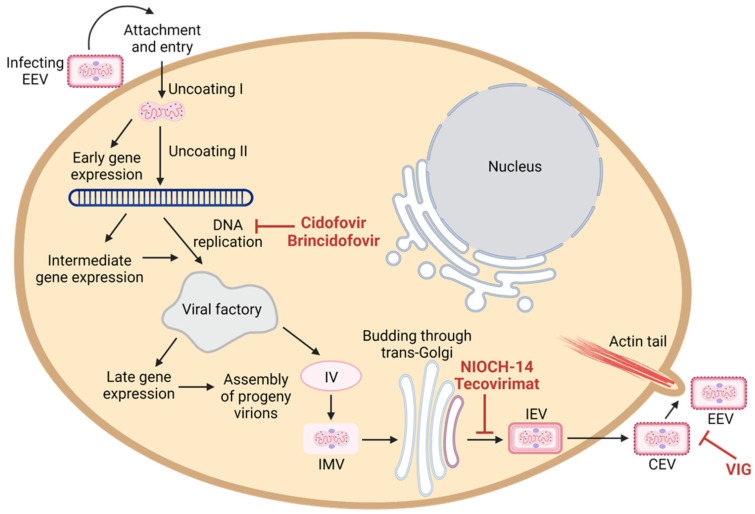
Schematic overview of the monkeypox virus life cycle and the mechanism of action of anti-poxvirus drugs. Like all poxviruses, monkeypox replicates in the cytoplasm of infected cells. Cidofovir and brincidofovir inhibit viral DNA polymerase; tecovirimat and NIOCH-14 prevents the formation of the cell-associated enveloped virion (CEV) and extracellular enveloped virion (EEV); and VIG prevents virion to infect new cells. The figure was created by Biorender.

Tecovirimat (ST-246 or TPOXX^®^) inhibits orthopoxviruses spreading in vitro by blocking p37 envelope protein, which plays a critical role in virus wrapping [78,79]. Specifically, tecovirimat prevents the formation of the cell-associated enveloped virion (CEV) and extracellular enveloped virion (EEV), which are two virion forms that are responsible for virus egress and dissemination (Figure 6) [80].

The antiviral potency of tecovirimat against monkeypox virus was evaluated in vitro and in various animal models. A submicromolar concentration of tecovirimat was shown to inhibit plaque formation of broad-spectrum orthopoxviruses, including monkeypox virus in cell culture assays [80,81,82]. The efficacy of oral administration of tecovirimat against monkeypox virus was shown in multiple animal models, including ground squirrels, prairie dogs, and nonhuman primates [78,83,84,85]. Non-human primates infected with a lethal dose of monkeypox indicated that a dose of 10 mg/kg initiated on day 4 or 5 post-infection for at least 7 consecutive days was sufficient to provide maximum survival rate and decrease in viral load [78]. Because smallpox was eradicated, the development and approval of tecovirimat were conducted under the under FDA Animal Rule 21 CFR 314 Subpart I [86]. Thus, the effective doses of tecovirimat in nonhuman primates that protect the animals from a lethal dose of orthopoxvirus infections were extrapolated and used for clinical trials [87]. A phase III clinical trial (NCT02474589) further validated the safety and pharmacokinetics of an oral regimen 600 mg twice daily for 14 days to a large group of human volunteers [78].

Tecovirimat is available as an oral (approved by FDA on 13 July 2018) and intravenous formulation (approved on 18 May 2022) to treat human smallpox diseases [88]. Oral formulation of tecovirimat (200 mg capsule) is approved for the treatment of smallpox, monkeypox, cowpox, and complications from vaccinia in adults and children weighing 13 kg and above by the European Medicines Agency (EMA) and FDA [89]. An injection formula was also licensed for smallpox disease in humans with a body weight of at least 3 kg [88]. Prior to receiving FDA approval, tecovirimat was used in combination with VIG and brincidofovir (CMX001) to treat severe eczema vaccinatum and progressive vaccinia under Emergency Investigational New Drug (EIND) application [90,91,92]. Treatment of tecovirimat in combination with VIG was given to a laboratory-acquired vaccinia virus infection patient [93] and generalized cowpox virus infection in an immunosuppressed patient [94]. Recently, tecovirimat has been used to treat monkeypox virus infection in the UK and the US [63,95]. There were no adverse events identified during the course of treatment, and notably, the patients in the UK had a shorter duration of symptoms and viral shedding compared to the other patients that had not received tecovirimat.

#### 7.3.2. Brincidofovir and Cidofovir

Cidofovir (CDV) is permitted for use as emergency investigational treatment in the case of a smallpox outbreak [96]. Intravenous-form Cidofovir (under the trade name Vistide) has been a licensed drug since 1996 for treating cytomegalovirus rhinitis in AIDS patients [97]. Cidofovir is a cytidine nucleotide analog that can interfere the viral DNA synthesis (Figure 6) [98]. However, there are limitations of cidofovir that may impede the use of cidofovir for poxvirus treatment. These include safety concerns due to nephrotoxicity [99], lack of oral bioavailability [100], and the compromised effect of cidofovir when given in combination with the smallpox vaccine [101].

Brincidofovir (BCV, CMX001, HDP-CDV, TEMBEXA^®^), a lipid-conjugated nucleotide analogue of cidofovir, has superior cellular uptake and conversion to the active form than cidofovir (Figure 6). The lipid moiety facilitates the cellular uptake of brincidofovir. Inside cells, cidofovir is released by cleavage activity of intracellular phospholipase enzymes and is converted into cidofovir diphosphate by kinases [102]. Brincidofovir counters two major limitations of cidofovir, in that brincidofovir is available in the form of an oral regimen and shows no evidence of nephrotoxicity [103]. The efficacy and safety of brincidofovir to treat orthopoxviruses are evaluated under the FDA Animal Rule [104]. Since the non-human primate model is not ideal for studying the efficacy of brincidofovir due to the rapid metabolism of brincidofovir into its inactive form [104], the efficacy of brincidofovir has been studied in other surrogate animal models for orthopoxviruses: mice infected with ectromelia virus, rabbits infected with rabbitpox virus, and prairie dogs infected with monkeypox virus [105,106,107]. Safety data of brincidofovir were also leveraged from the randomized phase 2 and phase 3 clinical trials against various DNA virus infections [108,109]. The notable mild adverse events of brincidofovir include gastrointestinal reactions, hepatotoxicity, elevations of liver enzymes ALT and AST, and elevation of total bilirubin. The proposed dosing regimen of oral brincidofovir is two doses of 200 mg once a week (two 100 mg tablets or 20 mL of suspension) to meet the acceptable safety profile for smallpox therapy [104].

On 4 June 2021, brincidofovir obtained approval from FDA for the treatment of smallpox in adults and pediatric patients, including neonates [110,111]. Brincidovofir (CMX001) was used in combination with VIG and tecovirimat to treat severe eczema vaccinatum and progressive vaccinia [90,91,92]. During the outbreak of monkeypox virus infection in the UK, three out of seven patients who had been treated with brincidofovir experienced elevations of liver enzymes (ALT and AST), and the treatment showed poor efficacy [63]. Considering the synergic effect of brincidofovir with tecovirimat [112] and approval to be used for treating pediatric patients, brincidofovir would complement tecovirimat in order to ensure a robust availability of therapeutics particularly in the presence of the tecovirimat-resistant virus or in case of a smallpox emergency.

#### 7.3.3. NIOCH-14

NIOCH-14 is a newly developed compound of tecovirimat analogue synthesized by the State Research Center of Virology and Biotechnology, Russia. This orally bioavailable compound demonstrated comparable results with tecovirimat studies in vitro, thus providing a promising candidate for the newer generation of anti-orthopoxvirus drugs [113].

#### 7.3.4. Vaccinia Immunoglobulin (VIG)

VIG is developed from pooled plasma collected from healthy donors who received a vaccinia vaccine and developed high titers of anti-vaccinia antibodies. The antibodies can bind to poxvirus virion and prevent the virus from infecting new cells (Figure 6). Two passive-immunization VIG intravenous (VIGIV) formulations have been approved by the FDA for the treatment of complications due to vaccinia vaccination (VIGIV Cangene and VIGIV Dynport; VIGIV product insert). The use of VIG for the treatment of severe infections with vaccinia was first introduced in 1960 [114]. Three clinical trials involving a total of 142 healthy male and female volunteers were conducted to evaluate the pharmacokinetic, pharmacodynamic, and safety profiles of VIGIV [115]. The VIGIV was shown to elicit mild adverse events when administered as single infusions of 6000 U/kg, 9000 U/kg, or 24,000 U/kg to healthy subjects. There was a lower incidence of adverse events when VIGIV was administered intravenously with the rate of infusion of 2 mL/min than 4 mL/min. A post-marketing clinical trial is underway to verify the clinical benefits of VIGIV for the treatment of complications due to vaccinia vaccination or vaccinia infections (NCT01374984). Coadministration of VIG and antiviral drugs have been used to treat severe eczema vaccinatum and progressive vaccinia [90,91,92,116] and other vaccinia vaccine complications [115]. The Centers for Disease Control and Prevention (CDC) has suggested that clinicians may consider VIG in severe cases of monkeypox. Prophylaxis in an exposed person who is contraindicated for smallpox vaccination is not yet officially indicated but may be offered [117].

## 8. Complications and Sequalae

Most monkeypox cases are entirely resolved within 2–4 weeks. However, some complications might occur following the infection. Encephalopathy and retropharyngeal abscess have been reported as severe complications [61]. Other complications have also been reported, including secondary skin infection, sepsis, bronchopneumonia, encephalitis, corneal infection, and deep abscess [63,118]. Pitted scarring was the most reported sequelae. Vision loss due to orbital infection was also reported in some cases. Severe complications and sequelae were more evident in nonvaccinated compared to vaccinated patients [43].

## 9. Prevention

Direct contact with the secretions of an infected person or animal, undercooked meat, or a contaminated object is the primary mode of viral transmission. The secretions can be respiratory droplets, skin or mucus membrane lesions, blood, or bodily fluid. As the recent outbreak in Europe and North America in 2022 has shown, which mainly affected men who have sex with men (MSM), there is a suggestion that monkeypox can be sexually transmitted, a fact that was previously unknown [119]. A recent study in Italy found viral DNA in the semen of infected patients persisting at least nine days after the onset of symptoms, although the proof of infectivity remains unclear [120]. Mother-to-child transmission via placenta or acquisition during or after birth has also been reported [76].

### 9.1. Prevention for Individual, Household, and Community

Hand hygiene is encouraged during the outbreak of monkeypox. The individual should avoid sharing personal items that may potentially harbor virus particles. For caretakers, maintaining the distance of at least one meter from the suspected or confirmed patient, wearing a mask that fits properly, and wearing disposable gloves are advised. Infected patients should remain in isolation and avoid close contact with any person or pet mammal until all skin lesions have crusted, scabs have come off, and a new layer of skin has formed underneath. However, MPXV may persist in bodily fluid even after all lesions have healed. Quarantine may be extended for up to 6 weeks after the last exposure to an infected person or animal [69]. For sexually active patients, the World Health Organization suggests using condoms for receptive or insertive sexual activity for 12 weeks after recovery [74].

A patient with monkeypox infection should be isolated at home in a well-ventilated space separate from other uninfected household members. If the patient needs assistance with self-care, the appointed person should be in good health without a high risk for severe monkeypox disease and should be vaccinated with smallpox. The caregiver should receive guidance regarding disease transmission and self-prevention.

Poxviruses can persist on household items, especially in dark, cool, and dry environments. Live viruses can be found retained in a patient’s residence for 15 days. Disinfectants should be applied to all areas that the infected patient occupies. A porous surface may contain live viruses for a longer duration than a nonporous one [121]. The patient’s clothing and bedding must be washed with soap and preferably at least 60 °C hot water. Shaking, dry dusting, sweeping, or vacuuming when cleaning home furnishings should be avoided to prevent the aerosolization of virus particles. The patient’s waste should be placed in a secured bag. Chlorine addition can also reduce contamination [74].

There is no evidence regarding the mode of delivery in infected pregnancy to prevent mother-to-child transmission. The indication for a cesarean section should conform to the general guideline. However, cesarean birth is recommended if any genital lesion is identified [122]. The baby born to an infected mother should be observed for symptoms and tested for viral DNA.

There is currently no evidence regarding the risk of viral transmission to the newborn during breastfeeding or the presence of viral antibodies in breastmilk. The practice should be assessed on a case-by-case basis weighing a risk and benefit calculation, taking into account the maternal status and severity of monkeypox disease.

### 9.2. Prevention and Control in Healthcare Settings

Contact and droplet precautions are implemented in confirmed cases. In addition to practicing hand hygiene, healthcare workers should wear personal protective equipment (PPE). Respirators are recommended as the evidence of airborne transmission of monkeypox is uncertain. Airborne precaution is recommended if aerosol-generating procedures are performed.

Instead of being quarantined, healthcare professionals exposed to monkeypox patients without adequate protection should undertake active surveillance for symptoms and have their temperature checked at least twice daily for 21 days after exposure [117].

The patient should wear a well-fitting medical mask, cover lesions, and be restricted in a well-ventilated isolation area. The confirmed case should maintain a minimum distance of at least one meter between patients. Severe cases or immunocompromised patients may have viral shedding prolonged in the respiratory secretion even after all the scabs have fallen off. A case-by-case evaluation may be required [74].

Previous and current evidence show unsatisfactory monkeypox knowledge among healthcare workers and students in health schools [123,124,125,126]. In addition, low levels of confidence to diagnose and manage monkeypox were shown in various settings [127,128]. This highlights the urgent need for educational and training intervention measures to help in the prevention and proper control of the ongoing outbreak.

In a recent review by Di Gennaro et al., the implementation of proper public health responses to contain the MPXV spread was delineated comprehensively [70]. Specifically, the specific actions at both the community and healthcare settings included: (1) vigilant surveillance for early detection and isolation of cases [70]; (2) training of healthcare workers to enable accurate and timely clinical diagnosis considering the current evidence of low self-reported confidence in the ability to diagnose and manage the disease among physicians and nurses [123,127,128]; (3) availability of accurate laboratory diagnostic kits cannot be overlooked, in light of the long list of differential diagnoses for patients presenting with unexplained acute rash with other nonspecific symptoms [70,71]; and (4) adherence to proper infection control measures, including the use of PPE and disinfection procedures [70].

## 10. Vaccines and Vaccination

The eradication of smallpox was one of the significant accomplishments of modern medicine and was accomplished through an effective vaccination program [129]. Following the eradication of smallpox in 1980, vaccination of the general population was discontinued after carefully considering the risks and benefits [130]. With nearly all children and most of the world population having little to no protection against orthopoxviruses, most people are vulnerable to the current monkeypox virus threat. Considering the escalating number of MPXV infection cases worldwide, the Advisory Committee on Immunization Practices (ACIP) recommended pre-exposure prophylaxis for health workers, laboratory personnel, clinical laboratory staff, and others who may be at risk of contracting the MPXV [131]. Here, we review the efficacy and safety of the ACIP-recommended vaccine against MPXV infections, including ACAM2000 and JYNNEOS.

Currently, the U.S. SNS contains more than 100 million doses of ACAM2000 and over 1000 doses of the JYNNEOS vaccine. Globally, the Smallpox Vaccine Emergency Stockpile (EVES) consists of approximately 2.4 million doses held by the WHO in Switzerland and more than 30 million doses pledged by several donor countries in case of international need [132].

### 10.1. ACAM2000

ACAM2000 is a replication-competent vaccinia virus vaccine used to generate the Dryvax vaccine, one of the earlier generations of vaccines used to eradicate smallpox [133]. The FDA licensed ACAM2000 in August 2007, and it was the only orthopoxvirus vaccine approved by FDA to prevent smallpox. ACAM2000 has been used for prophylaxis for those persons at high risk of exposure, including military personnel and research laboratory workers [134]. ACAM2000 is administered in a single dose percutaneously over the deltoid muscle through 15 jabs with a bifurcated needle, and a contagious lesion will develop at the site of this inoculation following successful vaccination [135].

Since human testing with either variola virus or MPXV is unethical, the efficacy of ACAM2000 was evaluated under the FDA Animal Rule in preclinical trials and by comparing the cutaneous/immunologic responses of ACAM2000 relative to Dryvax in clinical trial settings. The use of ACAM2000 in animal models (mice and cynomolgus macaques) showed that ACAM2000 is safer than Dryvax while still eliciting comparable cellular and humoral immunity [133]. ACAM2000 has demonstrated high levels of protection against monkeypox in cynomolgus macaques and prairie dog models [136,137,138]. Furthermore, the efficacy and safety of ACAM2000 have been evaluated in phase I, II, and III human clinical trials. More than 95% of vaccinia naïve subjects developed neutralizing antibody responses corresponding to the cutaneous responses [133,135,139].

The safety of the ACAM2000 was assessed in six clinical trials involving 2893 subjects who received ACAM2000 [134]. ACAM2000 vaccination can cause mild and severe adverse events, including progressive vaccinia, eczema vaccinatum, generalized vaccinia, inadvertent inoculation, encephalitis, myocarditis, and pericarditis [134,140,141]. Improved pre-vaccination screening for contraindications (e.g., individuals with immunocompromised states, atopic dermatitis, HIV infection, and allergies to the vaccine) could reduce the frequency and severity of serious adverse events [142]. Alternatively, persons with contraindications can be offered a vaccine with a more robust safety profile, such as an attenuated vaccinia virus vaccine.

### 10.2. JYNNEOS

JYNNEOS (also known as Imvamune or Imvanex) is a live attenuated vaccine derived from a replication-deficient modified vaccinia virus Ankara (MVA) [143] JYNNEOS was approved by the FDA in September 2019 for smallpox and monkeypox prevention in adults aged >18 [96]. JYNNEOS is administered in two doses of 0.5 mL four weeks apart through subcutaneous injection, with vaccine protection not conferred until two weeks after completion of the second dose [144]. Since JYNNEOS is a live attenuated virus that has lost the ability to replicate, there is no visible cutaneous response after vaccination and thereby no risk of spreading to other parts of the body or other people [131]. Therefore, it can be used for individuals with contraindications for a live replication-competent vaccine such as ACAM2000.

The efficacy of JYNNEOS against monkeypox has been assessed in animal model studies [136,138,143,145,146]. A phase III clinical trial is underway to assess its efficacy and safety against monkeypox in adult healthcare workers in the Republic of the Congo (NCT02977715) [15]. Phase II and Phase III clinical trials involving 22 studies with over 7000 subjects (healthy participants, HIV-positive volunteers, and people with atopic dermatitis or a history of atopic dermatitis) evaluated the efficacy and safety of the JYNNEOS vaccine [147,148,149,150,151,152,153]. JYNNEOS is considered to have a better cardiac safety profile, with no myocarditis or pericarditis being reported [152].

Considering overall improved safety profiles and efficacy of JYNNEOS have led ACIP to recommend the JYNNEOS vaccine as an alternative to ACAM2000 [131]. The JYNNEOS booster is recommended by ACIP every two years and ten years for those who work with virulent orthopoxviruses (smallpox and monkeypox) and less virulent orthopoxviruses (cowpox viruses), respectively [131]. Furthermore, ACIP recommends JYNNEOS boosters as an alternative to ACAM2000 for those who received ACAM2000 as the primary vaccine [131]. Currently, no data are available regarding the safety and efficacy of the JYNNEOS on special populations (e.g., children, pregnant women, and breastfeeding women). In the case of high-risk exposure, special populations may receive JYNNEOS in consultation with their health care provider after carefully weighing the risks and benefits.

## 11. Future Perspectives

The current monkeypox outbreak necessitated extensive epidemiological investigations, which pointed to a general lack of established travel links to endemic areas among the reported cases [1]. The available preliminary data showed that the ongoing 2022 monkeypox outbreak mainly involved MSM; however, the clustering of cases was not exclusive to this group [1,154,155]. Interpreting these preliminary epidemiologic investigation results requires special attention to avoid the potential attachment of stigma towards MSM [156].

The hope remains that the ongoing monkeypox outbreak can be contained [157]. However, such an objective requires vigilant surveillance, contact tracing, and raising the levels of knowledge and awareness, especially among health professionals [1,158,159]. This approach can help to improve the early detection of cases with subsequent termination of chains of transmission [160]. Previous and recent studies have shown that gaps in knowledge regarding monkeypox and the low confidence levels to diagnose, manage, and prevent the disease were widely prevalent among healthcare workers and university students in medical schools [124,127]. Therefore, this research area should be highlighted to help design strategies to properly control the ongoing outbreak and enhance preparedness for future anticipated epidemics.

In extreme situations, the investigative use of medicines that have shown to be beneficial against orthopoxviruses in animal trials and severe vaccinia vaccination effects may be something that should be examined. There is insufficient evidence to determine whether oral brincidofovir (DNA polymerase inhibitor), oral tecovirimat (intracellular viral release inhibitor), or the intravenous vaccinia immune globulin are effective against the MPXV [43]. Various preclinical studies have been conducted to find a potential treatment to cure monkeypox, and data on the safety and efficacy of these drugs are critical.

To produce a possible therapeutic antiviral agent, it is necessary to perform more in-depth studies on the genomic level and molecular analysis to shed light on host–viral interaction. Finally, clinical trials on potential treatment agents and vaccines are of utmost importance to control and prevent the current MPXV transmission.

## Figures and Tables

**Table 1:** Clinical symptoms reported in monkeypox infection.

Publication	Huhn et al. [61]	Pittman et al. [62]	Adler et al. [63]	Yinka-Ogunleye et al. [64]
Country	U.S.	Democratic Republic of the Congo	U.K.	Nigeria
Number of patients	37	216	7	122
Fever	87%	18.5%	42%	79%
Rash	97%	99.5%	100%	88%
Malaise	-	85.2%	-	50%
Myalgia	56%	6.9%	-	58%
Chill	71%	44.9%	-	65%
Adenopathy	71%	57.4%	71%	69%
Headache	65%	23.6%	-	79%
Sore throat	60%	78.2%	-	58%

## Data Availability

Not applicable.
